# Neutrophil and Monocyte CD64 and CD163 Expression in Critically Ill Neonates and Children with Sepsis: Comparison of Fluorescence Intensities and Calculated Indexes

**DOI:** 10.1155/2008/202646

**Published:** 2008-07-01

**Authors:** Mojca Groselj-Grenc, Alojz Ihan, Metka Derganc

**Affiliations:** ^1^Department of Paediatric Surgery and Intensive Care, University Medical Center, Zaloska 7, 1525 Ljubljana, Slovenia; ^2^Institute of Microbiology and Immunology, Faculty of Medicine, University of Ljubljana, Zaloska 4, 1000 Ljubljana, Slovenia

## Abstract

*Objective*. To evaluate the expression of CD64 and CD163 on neutrophils and monocytes in SIRS with/without sepsis and to compare the diagnostic accuracy of CD64 and CD163 molecules expression determined as (1) mean fluorescence intensities (MFI) of CD64 and CD163; and (2) the ratio (index) of linearized MFI to the fluorescence signal of standardized beads. *Patients and methods*. Fifty-six critically ill neonates and children with systemic inflammatory response syndrome (SIRS) and suspected sepsis, classified into two groups: SIRS with sepsis (*n* = 29) and SIRS without sepsis (*n* = 27). *Results*. CD64 and CD163 MFI measured on neutrophils and monocytes were elevated in patients with SIRS with sepsis. Diagnostic accuracy of indexes was equal to diagnostic accuracy of MFI for CD64 on neutrophils (0.833 versus 0.854 for day 0 and 0.975 versus 0.983 for day 1) and monocytes (0.811 versus 0.865 for day 0 and 0.825 versus 0.858 for day 1), and CD163 on neutrophils (0.595 versus 0.655 for day 0 and 0.677 versus 0.750 for day 1), but not for CD163 on monocytes. *Conclusion*. CD64 MFI, CD163 MFI, CD64 indexes for neutrophils and monocytes, and CD163 index for neutrophils can all be used for discrimination of SIRS and sepsis in critically ill neonates and children. CD64 index for neutrophils, however, is superior to all other markers.

## 1. INTRODUCTION

CD64 is a high-affinity and restricted isotype-specificity Fc*γ*RI receptor expressed on macrophages, monocytes, neutrophils, and eosinophils [1, 2]. There are several reports regarding its potential utility for the diagnostic assessment of sepsis or infection in adults [3–8] and neonates [9–12], but only a few in children [13, 14]. In an adult study, a higher intensity of CD64 expression has been found on neutrophils from patients with systemic inflammatory response syndrome (SIRS) and sepsis than on neutrophils from patients with SIRS only [3]. High CD64 expression on monocytes accompanying high CD64 expression on neutrophils has been reported in some adult studies as well [5, 15, 16].

CD163 is a monocyte/macrophage-associated antigen which has recently been identified as a haemoglobin scavenger receptor [17]. Apart from clearance of haemoglobin, it has also anti-inflammatory properties and an immunoregulatory role [18]. It has been found that CD163 expression on a monocyte surface is inversely related to the concentration of its soluble form (sCD163) in randomly selected patients [19]. During human experimental endotoxinemia, a rapid rise in plasma sCD163 has been observed together with reduced surface CD163 expression on isolated monocytes following lipopolysaccharide (LPS) administration, suggesting that LPS induces shedding of CD163 from the surface of isolated monocytes. Twenty-four hours after LPS administration in humans, CD163 surface expression is consistently increased over the baseline expression [20]. Soluble CD163 has been found to be elevated in sepsis in adults [21–23], whereas surface expression of membrane CD163 (mCD163) in sepsis has not yet been evaluated.

The first aim of the study was to evaluate the expression of CD64 and CD163 on neutrophils and monocytes in critically ill neonates and children with SIRS and sepsis and to find out whether it can discriminate between infectious and noninfectious SIRS. SIRS is most commonly caused by infection, but other conditions such as low-cardiac output syndrome, respiratory distress, and haemorrhage can trigger it in neonates and children. It has been found that as many as 82% of children admitted to a paediatric intensive care unit have two or more signs of SIRS [24].

The second aim of the study was to compare the diagnostic accuracy of CD64 mean fluorescence intensity (MFI), CD163 MFI, CD64 index of linearised MFI to standardised beads, and CD163 index of linearised MFI to standardized beads measured on neutrophils and monocytes. With recently available software for data analysis and index calculation, sources of technical errors and subjectivity, which are present in manual methods, are reduced and a comparison of results between different laboratories is possible [25].

## 2. PATIENTS AND METHODS

### 2.1. Patients and setting

This prospective observational study was conducted in the level III multidisciplinary neonatal and paediatric intensive care unit between January 2006 and September 2006. Fifty-six consecutive patients with SIRS and clinically suspected infections were eligible for enrolment. SIRS was defined according to the international pediatric sepsis consensus conference definitions [26]. Clinically suspected infection was defined as an explicit statement by the physician in the records, indicating the suspicion of an infection, combined with the initiation of diagnostic workup to rule out infection and the prescription of empirical antibiotic therapy. Patients were not included if they had received antibiotic therapy for more than 24 hours prior to admission, if they had undergone surgery in the previous week, or if they had a proven viral infection. The diagnosis of sepsis was confirmed by positive blood cultures or tracheal aspirates when chest radiographs showed signs of pneumonia. There were no other cultures (urine, cerebrospinal, or a puncture of normally sterile body fluid) positive in our patients. The diagnosis of clinical sepsis was established in patients with negative cultures, but with a strong suspicion of sepsis, who received a full course of antibiotic therapy. The group without sepsis included patients with a suspected infection in whom the subsequent clinical course, laboratory data, and microbiological tests excluded infection, and in whom antibiotic therapy was discontinued after a few days. Patients were classified into two groups: SIRS with sepsis and SIRS without sepsis. The classification was carried out by the attending physician unaware of the results of flow cytometry. We analyzed data separately for two age groups: neonates aged less than 28 days, and children older than 28 days, and for gram-positive and gram-negative sepsis. Patients' characteristics are summarized in Table 1. Pathogens isolated from blood cultures were *Staphylococcus epidermidis*(*n* = 3), *Streptococcus agalactiae*(*n* = 2), *Neisseria meningitidis*(*n* = 1), *Streptococcus pneumoniae*(*n* = 1) and *Streptococcus mitis*(*n* = 1), while pathogens isolated from tracheal aspirates were *Haemophilus influenzae*(*n* = 5), *Streptococcus pneumoniae* (*n* = 3), *Moraxella catarrhalis* (*n* = 2), *Escherichia coli* (*n* = 1), *Staphylococcus aureus* (*n* = 1), and *Pseudomonas aeruginosa* (*n* = 1).

The two groups were similar regarding gender (*χ* ^2^ test, *P* = 0.288), Pediatric Risk of Mortality Score (PRISM) III score (ANOVA, *P* = 0.835), age (ANOVA, *P* = 0.212 and *P* = 0.278 for neonates and children, resp.), gestational age (ANOVA, *P* = 0.839) and birth weight (ANOVA, *P* = 0.720).

The study was approved by the National Medical Ethics Committee of the Ministry of Health, Republic of Slovenia, and written consent was obtained from parents before blood sampling.

### 2.2. Sample collection and flow cytometry

**Figure 1:**
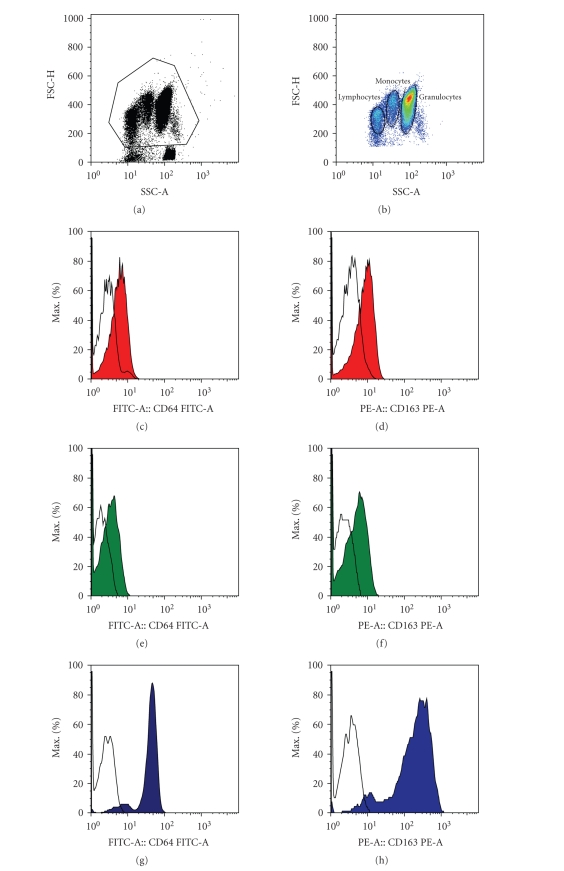
Gating of neutrophils, monocytes, and lymphocytes in FSC/SSC representative FACS diagrams. CD64 and CD163 histograms (colored) versus CD64 isotype controls (lined) are presented for granulocytes (red), lymphocytes (green), and monocytes (blue).

Blood samples (0.5 ml) were obtained at the time of suspected sepsis (day 0) and 24 hours later (day 1) together with samples for routine laboratory tests. Whole blood EDTA-anticoagulated samples were immediately transported to the flow cytometry laboratory during working hours or stored refrigerated (4°C) during the night or at weekends (up to 36 hours). Expressions of CD64 and CD163 on neutrophils, monocytes, and lymphocytes were measured by quantitative flow cytometry with a FACSCalibur flow cytometer (Becton Dickinson, NY, USA) using the Leuko64 assay (Trillium Diagnostics, LLC, Me, USA). The assay is for research use only and is composed of three antibodies with specificities to CD64 (clones 22 and 32.2, both fluorescein isothiocyanate (FITC) conjugated) and to CD163 (clone Mac2-148, phycoeritrin (PE) conjugated), and a fluorescence bead suspension with three fluorescence signals (green fluorescence due to FITC, orange fluorescence similar to PE, and red fluorescence of starfire red) for unique identification of beads, and used for instrument calibration and standardization of leukocyte CD64 and CD163 expression in human blood. The sample preparation and flow cytometer setup were based on the manufacturer instructions. Briefly, 50 *μ*L of whole blood, or diluted whole blood to adjust leukocyte concentration to less than 25 × 10^9^/L, was incubated for 15 minutes in the dark at room temperature with a mixture of murine monoclonal antibodies followed by red cell lysis with an ammonium-chloride-based red cell lysis solution (Trillium Lyse). Fluorescence beads were then added and flow cytometer analysis was performed on a minimum of 50 000 leukocytes. Data analysis for fluorescence intensity was performed by CellQuest software (Becton Dickinson, Calif, USA) (Table 2). MFI was measured as a linearized value of log scale on lymphocytes (red, negative control, measuring CD64 expression), monocytes (green, positive control, measuring CD64, and CD163 expression), neutrophils (blue, measuring CD64 expression), and beads (aqua blue, measuring FITC, and PE expression) (Figure 1). Index calculation was performed by Leuko64 QuantiCalc software (Trillium Diagnostics, Me, USA). Index measurements were derived by the ratio of linearized MFI of the cell population to the FITC signal from the beads. An internal negative control of the assay was provided by the automated measurement of the lymphocyte CD64 index, which had to be less than 1.0, and an internal positive control of the assay was provided by automated measurement of the monocyte CD64 index, which had to be more than 3.0. Flow cytometry was performed up to 36 hours after blood sampling. Before the beginning of the study the influence of delayed sample analysis was done and no significant difference in levels of CD64 and CD163 expression was detected in the first 36 hours after blood sampling. Isotype-control antibodies were routinely used in each experiment to detect nonspecific staining; however the calculation of CD64 and CD163 MFI was done without substracting isotype-control MFI in order to accurately compare the ratio (index) of linearized MFI to MFI alone.

### 2.3. Statistical analysis

Data were presented as the median and 95% confidence interval for the median. Comparison between groups was made using the unpaired Mann-Whitney test and analysis of variance (ANOVA). Proportions of patients were compared by the *χ* ^2^ test. Receiver-operating characteristic (ROC) curves were drawn to define the optimal sensitivity, specificity, cutoff value, and diagnostic accuracy, determined by the area under the ROC curve (AUC) of the studied surface antigens [27, 28]. The cutoff values at which the greatest sum of sensitivity and specificity was obtained were determined by the statistical program. Hanely and McNeil's comparison of diagnostic accuracies was performed. The CD64 score point (combination of two variables: CD64 MFI on neutrophils and monocytes or index CD64 for neutrophils and monocytes) was calculated for CD64 MFI and CD64 index, separately as described elsewhere [29]. When CD64 fluorescence intensity or CD64 index on both neutrophils and monocytes was under the cutoff level, the score point of 0 was assigned. The score point of 1 was assigned when the marker on one type of cells was under and marker on the other type of cells was over the cutoff level and the score point of 2 was assigned when markers on both types of cells were over the cutoff levels. The ROC curve analysis of CD64 score point was then performed. The differences were considered to be statistically significant at the level of *P* < .05. The statistical analysis was performed using Medcalc for Windows, version 5.0 (MedCalc Software, Mariakerke, Belgium) and Statistical Package for the Social Sciences for Windows, version 12.0 (SPSS Inc., Chicago, ILL, USA).

## 3. RESULTS

**Figure 2:**
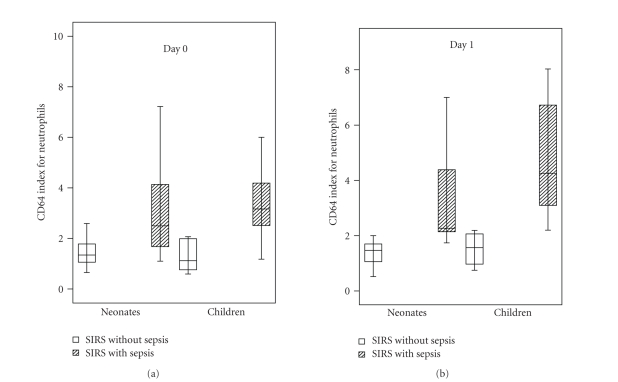
CD64 indexes for neutrophils at the time of suspected sepsis (day 0), and 24 hours later (day 1), for separate groups of neonates and children. Data are presented as box plots (median value and interquartile range). Outliers and extreme cases of index CD64 are not shown.

**Figure 3:**
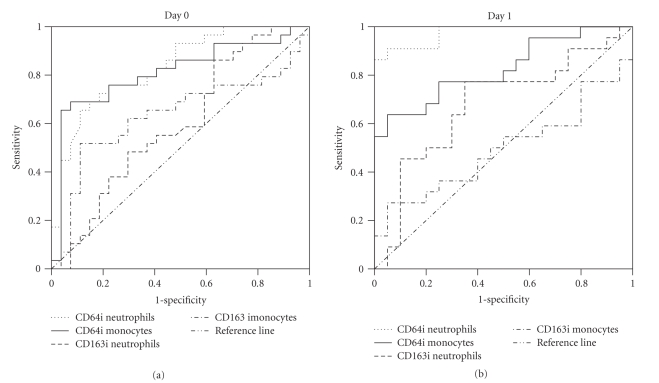
Receiver-operating characteristic (ROC) curves of CD64 and CD163 indexes (i) for neutrophils and monocytes for critically ill neonates and children with SIRS with sepsis and SIRS without sepsis at the time of suspected sepsis (day 0) and 24 hours later (day 1).

CD64 MFIs, CD163 MFIs, and CD64 indexes for both neutrophils and monocytes were significantly higher in patients with SIRS with sepsis compared with patients with noninfectious SIRS, while CD163 indexes showed statistical differences only on neutrophils on day 0 (Table 2). Statistically significant increase (*P* < .05) from day 0 to day 1 was found only for CD163 MFI for neutrophils in children with SIRS with sepsis and for CD163 MFI and index for monocytes in children with noninfectious SIRS. Medians of CD64 index for neutrophils were higher in children with sepsis than in neonates with sepsis, although differences were not significant (*P* > .05) at the time of suspected sepsis and 24 hours later (Figure 2). We did not find any statistically significant differences for CD64 and CD163 indexes, and CD64 and CD163 MFIs for neutrophils or monocytes, between gram-positive and gram-negative sepsis (*P* > .05 for all comparisons, data not shown). Optimum diagnostic cutoff levels, AUCs, sensitivity and specificity of CD64 and CD163 indexes, CD64 and CD163 MFIs for neutrophils and monocytes, and CD64 score points, for SIRS with sepsis at the time of suspected sepsis and 24 hours later are presented in Table 3. Setting sensitivity at more than 95% in ROC analysis of CD64 index for neutrophils displayed cutoff point 1.15 on day 0 (sensitivity 96.6% and specificity 40.7%) and 1.71 (sensitivity 100% and specificity 75.0%) on day 1. There were no significant differences between diagnostic accuracies of MFI and the corresponding index for either parameter, CD64 and CD163, on neutrophils and monocytes at the time of suspected sepsis and 24 hours later (*P* > .05 for all comparisons), except for diagnostic accuracy of CD163 MFI for monocytes, which was significantly higher at 24 hours than diagnostic accuracy of the CD163 index for monocytes (*P* = .003). The ROC curves of different indexes at the time of suspected sepsis and 24 hours later are presented in Figure 3. Diagnostic accuracies of CD64 indexes for neutrophils and monocytes at the time of suspected sepsis were both significantly higher compared with diagnostic accuracies of CD163 index for neutrophils (*P* = .000 and *P* = .023, resp.) and of CD163 index for monocytes (*P* = .028 and *P* = .050, resp.). Diagnostic accuracy of CD64 index for neutrophils at 24 hours was significantly higher compared with diagnostic accuracies of CD64 index for monocytes (*P* = .009), CD163 index for neutrophils (*P* = .000), and CD163 index for monocytes (*P* = .000). The combinations of two variables: CD64 MFI for neutrophils with CD64 MFI for monocytes or CD64 index for neutrophils with CD64 index for monocytes (CD64 score point) insignificantly increased diagnostic accuracy of CD64 MFI (*P* = .559) or CD64 index (*P* = .534) for neutrophils on day 0, while there was an insignificant fall in diagnostic accuracy (*P* = .494 and *P* = .128, resp.) on day 1 (Table 3).

## 4. DISCUSSION

To our knowledge, this is the first study which has evaluated expression of leukocyte antigens CD64 and CD163 in critically ill neonates and children with SIRS with sepsis. In this study, we have shown that expression of CD64 and CD163 on neutrophils and monocytes is elevated in patients with sepsis compared with patients with noninfectious SIRS. We have also shown that diagnostic accuracy of computer-calculated indexes for CD64 on neutrophils and monocytes and CD163 on neutrophils based on the ratio (index) of linearized MFI to the fluorescence signal of standardised beads, is equal to the diagnostic accuracy of manually determined MFI, if proceeded by the same flow cytometer. Our results therefore suggest that the cost-effectiveness of tests, based on multiple antibodies (CD64 and CD163) and standardized beads should be reevaluated.

CD64 is expressed at low concentration on the surface of nonactivated neutrophils [30]. CD64 surface upregulation is induced by granulocyte colony-stimulating factor (G-CSF) and interferon-*γ* (INF-*γ*) within 4–6 hours of stimulation [25]. Our study confirmed the results from studies of the adult population that the level of CD64 expression is significantly higher in patients with SIRS with sepsis compared with patients with noninfectious SIRS [3]. In our study, the expression of CD64 on neutrophils was significantly higher in critically ill neonates and children with SIRS with sepsis compared with those with noninfectious SIRS. So far, CD64 expression on leukocytes has been studied mostly in the neonatal population. These studies showed that CD64 expression on neutrophils is upregulated in early-neonatal infection [9, 10], late-neonatal infection [11, 12], and in preterm neonates with infection [11, 13]. Some authors have even suggested that, because of its high sensitivity, measurement of neutrophil CD64 expression may allow clinicians to discontinue antimicrobial treatment if negative within 24 hours of suspected infection, without waiting for the definitive microbiological results [9]. The results of our research are in accordance with the results of this other study [9] only to some extent. The AUCs in both studies were similar, but in the other study [9], higher sensitivity was achieved, probably because the cutoff values were determined in advance to enable better sensitivity with the aim to identify all infected cases. Due to the different method of flow cytometry used for the measurement of CD64 expression in the other study [9], the cutoff values cannot be compared. In our study the cutoff values were determined by the statistical program to obtain the greatest sum of sensitivity and specificity for comparisons of different markers. In order to increase the sensitivity we determined the cutoff values at which the sensitivity was 96.6% and 100%, respectively, on day 0 and 1. The specificity was then slightly lower on day 1 than that in the previously mentioned study [9]. Three cases of sepsis caused by coagulase-negative staphylococci could have lowered the sensitivity of CD64 expression on neutrophils in our study, whereas in the other study [9] there were no such cases. In children, there are only two reports of 14 infants and children, and 8 children, respectively, hospitalized because of different bacterial infections (mostly pneumonias and pyelonephritides) in whom CD64 expression on neutrophils was significantly higher compared with noninfected controls [13, 14]. So far, CD64 expression has not been studied in septic critically ill children. In adult patients, higher expression of CD64 on neutrophils was found in gram-negative sepsis compared with gram-positive sepsis [15]. In the recent study in neonates [31], as in our study, this difference was not confirmed. The cause for this difference between adults and neonates could be a less expressed neutrophil response to infection with gram-negative bacteria in neonates. Indeed, we found lower CD64 index in neonates with sepsis than in children, although the difference was not statistically significant. A novel marker of infection, the CD64 score point, which incorporates the quantitative analysis of CD64 expression on both neutrophils and monocytes has recently been introduced as a marker in adults which could distinguish between infection and healthy states [29]. In our study, the CD64 score point was able to discriminate between SIRS with sepsis and noninfectious SIRS as well, although its diagnostic accuracy was not higher than that of CD64 expression on neutrophils themselves.

To our knowledge, CD64 expression on monocytes in neonates and children has not previously been studied. There are some reports of elevated CD64 expression on monocytes in adult patients with sepsis [5, 15, 16]. In our study, the results of CD64 on monocytes and neutrophils were similar at the time of suspected sepsis, but 24 hours later the diagnostic accuracy of CD64 on monocytes was significantly lower than on neutrophils. This data could indicate that activation in monocytes is faster or more rapidly completed than in neutrophils [32].

The increased expression of CD163 is a part of the maturation of a monocyte to a phagocytic macrophage [18]. The expression of CD163 is upregulated by interleukin-6 and glucocorticoids together with interleukin-10, and downregulated by LPS and interferon-*γ* [18]. Although the absolute value of median expression of CD163 was much lower on neutrophils than on monocytes in our patients with sepsis, the expression of CD163 on both types of cells could differentiate between patients with SIRS with sepsis and noninfectious SIRS in our study. As dynamics of fluorescence intensity for CD64 and CD163 on neutrophils was diagnostically very significant, it is highly unlikely that a nonspecific staining would have been the cause. Also, the representative FACS diagrams made possible quite a sharp distinction between cell populations without causing serious problems in cell gating. Our results therefore indicate that, like monocytes, neutrophils are able to some extent to express CD163.

Membrane CD163 expression has not yet been clinically evaluated. Only one author found increased bone marrow infiltration with CD163-positive macrophages in postmortem analysis of samples from patients who died from severe sepsis or septic shock compared with controls [33]. In contrast, high concentrations of soluble CD163 (sCD163) have been described in adult septic patients in several studies [3, 21]. It has been shown experimentally with cultured monocytes that CD163 can be shed from the cell membrane after LPS stimulus [20], cross-linking of the Fc receptor for immunoglobulin G [34] or oxidative stress [35]. The shedding of CD163 from monocyte membrane correlates with decreased expression of membrane CD163 on cultured monocytes [20, 34]. The decreased expression of membrane CD163 is probably partly due to shedding of CD163 [20] and partly due to decreased CD163 mRNA synthesis [34, 36]. In healthy adult volunteers, CD163 surface expression was increased again 24 hours after LPS stimulation [20]. The precise time point of increased expression is not yet exactly known, but it was estimated to be 0–24 hours after LPS stimulation in adult volunteers [20] or even later in cultured monocytes [34]. It was shown that stimulation with LPS induced short-term suppression of CD163 mRNA expression in cultured monocytes, while long-term cultures of monocytes treated with LPS showed intereleukin-10 dependent recovery of surface CD163 expression [34]. In our clinical study, we did not observe the transitional fall of membrane CD163 expression on monocytes. The expression of CD163 on both monocytes and neutrophils was higher in septic patients than in patients with noninfectious SIRS both at the time of suspected sepsis and 24 hours later. The reason may be that clinical signs are observed with significant delay after endotoxinemia.

Most clinical studies in the past have used manually determined fluorescence intensity to describe CD64 expression on neutrophils. This method, however, is subject to day-to-day operational and instrumental fluctuations and cannot be used for comparison between different laboratories. The newly developed method for computer-calculated indexes uses calibration beads which serve as an internal fluorescence intensity standard and, together with fully automated software for index calculation, which removes the subjectivity in data analysis by the end users, provide a novel approach in minimizing lot-to-lot variation, and in removing the subjectivity due to different range of experience and skills of operators [25]. In our study, comparison of manually determined fluorescence intensity and computer-calculated index revealed that diagnostic accuracy of both parameters was equal for CD64 on neutrophils and monocytes and CD163 on neutrophils, but not for CD163 on monocytes. Our explanation is that automated index calculation was originally developed for evaluation of CD64 index on neutrophils in different clinical conditions [25] and the method is probably not universally transmittable to evaluate all other antigens on different cell types for diagnostic purposes. The calculation of CD64 index for neutrophils, which achieved the highest diagnostic accuracy in our study, was optimal and we suggest that this parameter should be used in future evaluations. We found no studies, except for a poster presentation at Sepsis 2007 in Paris [37], which have used index calculation for evaluation of CD64 expression on neutrophils.

Some limitations of this study merit consideration. Firstly, the definition of clinical sepsis with culture-negative patients, particularly critically ill neonates, is still a matter of discussion. Secondly, the number of patients is relatively small. A greater number of neonates and children is needed for a more precise evaluation of diagnostic accuracies of selected infection markers (CD64 and CD163 index for monocytes and neutrophils) and for comparison with routinely used infection markers in the future. The blood samples were processed within 36 hours in our study. For clinical use, the method should be available every day, particularly because some authors suggest processing of blood specimens for analysis of cell surface markers immediately after obtaining them to avoid cell apoptosis [30]. However, other authors claim that measurements for neutrophil CD64 assay are stable for up to 30 hours at room temperature and up to 72 hours refrigerated [25]. As we already mentioned, no significant differences in levels of CD64 and CD163 expressions were obtained in the first 36 hours after blood sampling in our study.

## 5. CONCLUSION

The data from the present study show that CD64 and CD163 expressions on neutrophils and monocytes are elevated in critically ill neonates and children with SIRS with sepsis. Diagnostic accuracies of computer-calculated indexes for CD64 on neutrophils and monocytes and CD163 on neutrophils based on the ratio (index) of linearised MFI to the fluorescence signal of standardized beads are equal to diagnostic accuracy of manually determined MFI, if processed by the same flow cytometer. Therefore, CD64 MFI, CD163 MFI, CD64 indexes for neutrophils and monocytes and CD163 index for neutrophils can all be used for discrimination of SIRS and sepsis in critically ill neonates and children. CD64 index for neutrophils, however, is superior to all other markers.

## Figures and Tables

**Table 1:** Characteristics of the study population.

	SIRS with sepsis	SIRS without sepsis
Number	29	27
Male/female	12/17	16/11
Median PRISM III (range)	11 (4–26)	12 (2–23)
Number of deaths	0	3 (11%)
Number of mechanically ventilated patients	27 (93%)	27 (100%)
Number of patients with severe sepsis	16 (55%)	/
Number of patients with septic shock	12 (41%)	/
Number of patients with inotropic drugs	12 (41%)	9 (33%)

Number of neonates <28 days	15	22
Number of newborns (0–7 days)	13	20
Median age (range); days	1 (0–18)	0 (0–13)
Median gestational age (range); weeks	38.2 (32.4–41.0)	37.9 (32.4–41.4)
Median birth weight (range); g	2945 (1400–3850)	3025 (1350–3920)

Number of children >28 days	14	5
Median age (range); months	9.3 (1.9–33.0)	4.2 (1.4–65.9)

Number of gram-positive sepsis	11	/
Number of gram-negative sepsis	10	/
Number of culture-negative sepsis	8 (28%)	/
Number of positive blood cultures	8 (28%)	/
Number of positive tracheal aspirates	13 (44%)	/

**Table 2:** Median CD64 and CD163 MFI and indexes (i) for neutrophils and monocytes with 95% confidence interval (CI) in critically ill neonates and children at the time of suspected sepsis (day 0) and 24 hours later (day 1).

	Day 0			Day 1		
	SIRS with sepsis	SIRS	*P*	SIRS with sepsis	SIRS	*P*
Neutrophils						
CD64f_MFI_	83 (66–108)	44 (36–56)	.0000*	100 (75–162)	47 (32–54)	.0000*
CD64i	2.65 (2.02–4.03)	1.3 (1.06–1.78)	.0000*	3.36 (2.27–4.68)	1.47 (1.07–1.78)	.0000*
CD163_MFI_	65 (50–85)	47 (44–64)	.0463*	80.5 (57–115)	50.5 (40.2–84.8)	.0056*
CD163i	456 (423–548)	447 (381–491)	.2250	527 (486–621)	462 (426–528)	.0495*

Monocytes						
CD64_MFI_	251 (231–294)	168 (149–212)	.0000*	307 (257–354)	195 (159–243)	.0001*
CD64i	9.36 (7.94–11.2)	6.69 (5.6–7.28)	.0001*	11 (8.39–13.4)	7.41 (6.43–8.22)	.0003*
CD163_MFI_	2350 (1674–3942)	1451 (1234–1637)	.0063*	3281 (2369–4093)	2483 (1927–2937)	.0439*
CD163i	20080 (9330–27352)	9460 (7708–14893)	.0836	21136 (13636–32551)	20142 (15926–25366)	.9598

*statistically significant differences, Unpaired Mann-Whitney test.

**Table 3:** Optimum diagnostic cutoff level, diagnostic accuracy with 95% confidence interval (CI) determined by the area under the ROC curve (AUC), sensitivity, and specificity for given cutoff levels of CD64 and CD163 MFI and indexes (i) for neutrophils and monocytes and CD64 score points for sepsis prediction in critically ill neonates and children at the time of suspected sepsis (day 0) and 24 hours later (day 1).

	Cutoff level		AUC (95% CI)		Sensitivity (%)		Specificity (%)	
Day	0	1	0	1	0	1	0	1
CD64_MFI_ for neutrophils	72	65	0.854 (0.734–0.934)	0.983 (0.885–0.995)	65.5	95.5	92.6	95.0
CD64i for neutrophils	2.45	2.19	0.833 (0.709–0.919)	0.975 (0.872–0.996)	65.5	86.4	88.9	100
CD64_MFI_ for monocytes	228	266	0.865 (0.747–0.941)	0.858 (0.715–0.946)	72.4	72.7	88.9	95.0
CD64i for monocytes	8.70	9.47	0.811 (0.684–0.903)	0.825 (0.677–0.924)	65.5	63.6	96.3	95.0
CD163_MFI_ for neutrophils	47	55	0.655 (0.516–0.777)	0.750 (0.592–0.870)	75.9	86.4	51.9	60.0
CD163i for neutrophils	391	482	0.595 (0.455–0.724)	0.677 (0.515–0.813)	86.2	77.3	37.0	65.0
CD163_MFI_ for monocytes	1641	3033	0.713 (0.576–0.826)	0.682 (0.520–0.817)	75.9	63.6	74.1	85.0
CD163i for monocytes	19257	34282	0.635 (0.495–0.759)	0.500 (0.338–0.654)	51.7	27.3	88.9	95.0
CD64_MFI_ score point	>0 (1 and 2)	>0 (1 and 2)	0.879 (0.764–0.951)	0.961 (0.851–0.995)	86.2	100.0	85.2	90.0
CD64i score point	>0 (1 and 2)	>0 (1 and 2)	0.864 (0.746–0.941)	0.914 (0.785–0.977)	82.8	90.9	88.9	80.0
